# Evidence for Loss of a Partial Flagellar Glycolytic Pathway during Trypanosomatid Evolution

**DOI:** 10.1371/journal.pone.0103026

**Published:** 2014-07-22

**Authors:** Robert W. B. Brown, Peter W. Collingridge, Keith Gull, Daniel J. Rigden, Michael L. Ginger

**Affiliations:** 1 Faculty of Health and Medicine, Division of Biomedical and Life Sciences, Lancaster University, Lancaster, United Kingdom; 2 Sir William Dunn School of Pathology, University of Oxford, Oxford, United Kingdom; 3 Institute of Integrative Biology, University of Liverpool, Liverpool, United Kingdom; University of Hull, United Kingdom

## Abstract

Classically viewed as a cytosolic pathway, glycolysis is increasingly recognized as a metabolic pathway exhibiting surprisingly wide-ranging variations in compartmentalization within eukaryotic cells. Trypanosomatid parasites provide an extreme view of glycolytic enzyme compartmentalization as several glycolytic enzymes are found exclusively in peroxisomes. Here, we characterize *Trypanosoma brucei* flagellar proteins resembling glyceraldehyde-3-phosphate dehydrogenase (GAPDH) and phosphoglycerate kinase (PGK): we show the latter associates with the axoneme and the former is a novel paraflagellar rod component. The paraflagellar rod is an essential extra-axonemal structure in trypanosomes and related protists, providing a platform into which metabolic activities can be built. Yet, bioinformatics interrogation and structural modelling indicate neither the trypanosome PGK-like nor the GAPDH-like protein is catalytically active. Orthologs are present in a free-living ancestor of the trypanosomatids, *Bodo saltans*: the PGK-like protein from *B. saltans* also lacks key catalytic residues, but its GAPDH-like protein is predicted to be catalytically competent. We discuss the likelihood that the trypanosome GAPDH-like and PGK-like proteins constitute molecular evidence for evolutionary loss of a flagellar glycolytic pathway, either as a consequence of niche adaptation or the re-localization of glycolytic enzymes to peroxisomes and the extensive changes to glycolytic flux regulation that accompanied this re-localization. Evidence indicating loss of localized ATP provision via glycolytic enzymes therefore provides a novel contribution to an emerging theme of hidden diversity with respect to compartmentalization of the ubiquitous glycolytic pathway in eukaryotes. A possibility that trypanosome GAPDH-like protein additionally represents a degenerate example of a moonlighting protein is also discussed.

## Introduction

Glycolysis describes the catabolism of glucose to two molecules of pyruvate. The pathway requires the successive activities of ten enzymes, and results in the net production of two molecules of ATP and the reduction of two molecules of NAD^+^ per molecule of catabolized glucose. In many cells, glycolytic flux contributes the major or even sole source of metabolic energy, and in eukaryotes glycolysis is classically considered a ‘cytosolic’ pathway. Yet, in response to appropriate extrinsic or intrinsic cues cytosolic glycolytic enzymes from various animals, plants, yeast and protists form cytoskeleton- or organelle-associated multi-protein complexes [1]–[5]. As exemplified by studies of plant cells, where dynamic re-localization of glycolytic enzymes to the outer-mitochondrial membrane occurs as a function of respiratory activity, enzyme re-localization facilitates channeling of pathway intermediates between sequential glycolytic enzymes without equilibration with the bulk solution phase of the cytosol occurring [3]. In plant cells, this likely directs glycolysis-derived pyruvate towards mitochondrial metabolism, rather than the provision of precursors for competing cytosolic pathways.

Aside from plants and algae, where glycolytic enzymes are also used in plastids for carbon fixation through the Calvin cycle and in the provision of precursors for plastid-localized biosynthetic pathways, the ‘classic’ paradigm of glycolysis as a cytosolic pathway is also challenged by observations of glycolytic enzyme targeting to the mitochondrial matrix [6]–[8], peroxisomes [9]–[11] and flagella (or cilia, terms referring to essentially the same organelle) [12],[13]. An extreme example of glycolytic enzyme compartmentalization is seen in kinetoplastid protists, a cosmopolitan group of flagellates that include the parasitic trypanosomatids, which are responsible for the tropical diseases African sleeping sickness, Chagas' disease and leishmaniasis. In these protists, depending upon the species and life cycle stage examined, either the first six or the first seven glycolytic enzymes are targeted to peroxisomes, but are absent from the cytosol. As a consequence trypanosomatid peroxisomes are aptly better known as glycosomes [10]. Intriguingly, a recent report of peroxisomal targeting for some glycolytic enzymes in a wide variety of fungi and the prediction of peroxisomal 3-phosphoglycerate kinase (PGK) targeting in mammalian cells suggests peroxisomal partitioning of a partial glycolytic pathway may be more common than hitherto thought [9], although the use of alternative splicing and stop codon read-through to generate peroxisomal and cytosolic isoforms of glycolytic enzymes in fungi, and potentially animals, is very different to the exclusively peroxisomal localization of glycolytic enzymes seen in trypanosomatids.

The regulation of glycolysis is also different in trypanosomatids, as compared with other organisms, in that feedback inhibition of neither hexokinase nor phosphofructokinase is perceived as important for pathway regulation; indeed many of the mechanisms which stimulate or inhibit the activity of these enzymes in other eukaryotes are absent in trypanosomes (reviewed in [14] and see also [15]–[17]). The available data, obtained mostly from modelling and experimental analysis of the African sleeping sickness parasite *Trypanosoma brucei*, indicate that in an apparent absence of regulatory controls acting on hexokinase and phosphofructokinase activities, glycosomal compartmentalization of glycolytic enzymes protects the parasite from toxic accumulation of glycolytic intermediates [16]–[20]. Peroxisomes (and glycosomes) are closed compartments with respect to an easy exchange of ATP and ADP; hence, a consequence of the unregulated phosphorylation of glucose and fructose-6-phosphate is a requirement to ensure efficient re-generation of intraglycosomal ATP. In bloodstream stage *T. brucei*, a glycosomal PGK regenerates ATP hydrolyzed inside the glycosome during the activation of glucose to fructose-1, 6-bisphosphate. Lethality arising from ectopic expression of cytosolic PGK activity in bloodstream *T. brucei* provides experimental support for this assertion [21], and this lethal phenotype can be understood in terms of channels in the glycosomal membrane that select on a basis of size and facilitate free diffusion of glycolytic intermediates between glycosomal matrix and the cytosol (in contrast to apparent restricted exchange of ATP and ADP) [22]. Thus, in mutants analyzed by Blattner et al. (1998) there is competition between the native glycosomal PGK and ectopic cytosolic PGK for the substrate 1,3-bisphosphoglycerate, which diffuses between glycosome and cytosol. As a consequence, failure to restore glycosomal ATP at a rate that sustains glycolytic flux provides an explanation for cell death [21]. In procyclic stage *T. brucei* (the life cycle stage that replicates in the mid-gut of the tsetse fly vector), measurable PGK activity is mostly detected in cytosolic fractions [23]. Here, up-regulation of glycosomal isoforms of adenylate kinase, pyruvate phosphate dikinase, and phospho*enol*-pyruvate carboxykinase provide alternative enzymes to PGK for maintaining intraglycosomal homeostasis of adenine nucleotide concentrations [10]. Glucose is not considered to be an abundant carbon source within the digestive tract of the parasite's tsetse fly vector and the up-regulation of the fore-mentioned enzymes is therefore explained, at least in part, by the participation of glycolytic enzymes and glycosomes in the energy-consuming pathway of gluconeogenesis.

Here, we describe *T. brucei* flagellar proteins homologous to the glycolytic enzymes glyceraldehyde-3-phosphate dehydrogenase (GAPDH) and PGK, and suggest that these proteins represent a relic of a flagellar glycolytic pathway that degenerated during trypanosomatid evolution.

## Materials and Methods

### Bioinformatics

Homologs of *T. brucei* GAPDH-like and PGK-like sequences, both members of the same inactivated families and active enzymes, were found using local BLAST [24] searches of the TriTryp database release 6 [25]. GAPDHL and PGKL sequences were aligned against selected catalytic sequences using MUSCLE [26] and the results manipulated and viewed with Jalview [27]. The comparison sequences were obtained via a BLAST search of the UniRef50 [28] low-redundancy sequence database (GAPDHL), or from a Reference Proteome 15 [29] set downloaded from Pfam [30] and filtered at a 40% sequence identity level using CD-HIT [31] (PGKL). Sequence conservation was mapped to model structures using Consurf [32]. Phylogenetic analysis was carried out on GAPDHL and PGKL sequence alignments using the MEGA 5 software [33] to generate trees by protein distance-based Neighbor-Joining [34], Minimum Evolution [35] and Maximum Likelihood based on the JTT matrix-based model [36]. Gapped positions were not considered in the calculations and bootstrapping analysis (500 replicates; [37]) was done to estimate confidence in nodes. Results from the phylogenetic analyses were used to infer the orthology of BS06470 with *Tb*GAPDHL. Sequence identities between families were calculated from the alignments using MODELLER [38]. Prediction of transmembrane helices was done with TMHMM [39] and Phobius [40]. The Pfam database [30] was used to analyze the phylogenetic distribution of protein domains of interest.

The HHPred server [41] was used to clarify the existence of the C-terminal domains in PGKL, to obtain automated models of PGKL domains and of *B. saltans* GAPDHL, and to rank templates for homology model building of *Tb*GAPDHL. More rigorous modeling of *Tb*GAPDHL was subsequently carried out locally, building with MODELLER [41] and selecting according to both packing (DOPE score; [42]) and stereochemical quality (Ramachandran plot calculated with Procheck [43]). PyMOL (http://pymol.org) was used to visualize and present the results.

### Cell culture

Procyclic *T. brucei* (S427 and 927smox [44]) were cultured in SDM-79 medium supplemented with 10% heat-inactivated fetal calf serum and hemin. Logarithmic phase cultures (at densities of ∼5×10^6^–10^7^ cells ml^−1^) were stably transformed using standard approaches [45], with selectable markers used at the following final concentrations: phleomycin, 3 µg ml^−1^; blasticidin S, 10 µg ml^−1^; puromycin, 2 µg ml^−1^; hygromycin 50 µg ml^−1^.

### Expression and localization of epitope-tagged *Tb*GAPDHL and *Tb*PGKL

For expression of GFP::*Tb*GAPDHL, YFP::*Tb*GAPDHL, Ty::*Tb*PGKL from endogenous gene loci pEnT-based vectors [46] were used. For GFP::GAPDHL and YFP::GAPDHL expression bp +4 to +386 of the Tb*GAPDHL* coding sequence and bp −252 to −236 from the 5′ intergenic sequence were cloned into pENT5-G and pENT6B-Y, respectively. GFP::*Tb*GAPDHL was expressed in procyclic S427 *T. brucei* and YFP::*Tb*GAPDH was expressed in *Tb*CAM [47] and *snl-2* [48] RNAi backgrounds. For expression of Ty::*Tb*PGKL, bp +4 to +371 of the Tb*PGKL* coding sequence and bp −382 to −330 from the 5′ intergenic sequence were cloned into pENT6P. Ty::*Tb*PGKL was expressed in procyclic S427 *T. brucei*. All DNA constructs were sequenced using ABI Prism sequencing technology. Expected molecular masses of the expressed fusion proteins were confirmed by immunoblotting using standard methods; anti-GFP antibody preparation (mixed monoclonal antibodies 7.1 and 13.2, Roche) was used as per the manufacturer's instructions, and the BB2 monoclonal antibody as described previously [49].

For microscopy, live cells were settled onto coverslips and either fixed with 3.7% paraformaldehyde or extracted for 45 sec with 0.1% Nonidet-P40 in 0.1 M PIPES, 2 mM EGTA, 1 mM MgSO_4_, 0.1 mM EDTA, pH 6.9 (yielding cytoskeletons) prior to paraformaldehyde fixation. Indirect immunofluorescence with L8C4, L3B2, and BB2 antibodies was carried out as described previously [50], [51]. Images were captured using an Applied Precision DeltaVision Deconvolution microscope system and processed using SoftWoRx software and finally formatted using Adobe Photoshop.

### Gene disruption of *PGKL* and *GAPDHL*

For disruption of *PGKL* (encoded by Tb927.11.2380) from diploid *T. brucei*, blasticidin deaminase or phleomycin-resistance genes flanked by tubulin and actin mRNA processing signals were amplified by PCR from pCP101 or pRM481 templates, respectively [52], [53], using the primer combination 5′-atgtctcttagcgccttacggtccaaacgctgggtcccattgtttgcctc-3′ and 5′-tgcgttaataccctctattttgttactcggtattttatggcagcaacg-3′. Purified PCR products were then used as templates for a second PCR amplification using primer combination 5′-taccacatataaagaaaaaagtttcccgccatgtctcttagcgcttacg-3′ and 5′-gggtttgggcatgtgttttttcctgaaatatgcgttaataccctctattt-3′. In this way, PCR products from the second reaction now contained genes capable of conferring resistance to either blasticidin S or phleomycin, and flanked upstream by a homology targeting flank corresponding to bp −30 to +30 bp of Tb*PGKL* and downstream by a homology flank corresponding to the 13 bp upstream and 44 bp downstream of the stop codon for Tb*PGKL*. ∼5 µg of each PCR product was used independently for stable transformation of procyclic *T. brucei*, and disruption of into one *PGKL* allele via homologous recombination. Genomic DNA was isolated from Tb*PGKL* ^+/−^ heterozygotes as described previously, and the correct integration of blasticidin deaminase and phleomycin-resistance genes confirmed by Southern blotting and PCR. For the PCR using Tb*PGKL* ^+/−^ templates the primer combination 5′-cttagttgcataatgcccacc-3′ and 5′-cctttagcgcaaatcgagtcc-3′ was used; amplification from an endogenous Tb*PGKL* allele yielded a PCR product of ∼3.7 kb, but from alleles in which the PGKL gene had been disrupted by integration of either the blasticidin deaminase or the phleomycin-resistance gene PCR products of ∼2 kb were obtained. In this way, drug-resistance genes containing an upstream homology flank of 534 base pairs and a downstream homology flank of 499 base pairs were generated; the longer homology targeting flanks were used for the disruption of the remaining Tb*PGKL* allele in reciprocal transfections of heterozygote cell lines – *i.e.* phleomycin-resistant Tb*PGKL* ^+/−^ was transfected with the blasticidin deaminase gene adjoined by long homology flanks or vice-versa.

Essentially the same transfection strategy was used for the generation of Tb*GAPDH* ^−/−^ mutants. For initial amplification of blasticidin and phleomycin resistance-conferring genes, a primer combination of 5′-cgaaggtatactgatggaggcggaaacgagtgggtcccattgtttgcctc-3′ and 5′-atctctttcctgtggcaaacgtcaaggccgtattttatggcagcaacg-3′ was used. Using the resultant PCR amplicons as templates, subsequent PCR reactions (one for each drug-resistance gene) were carried out using a primer combination of 5′-gcacaaacagggaagccgtacggaaccgcccgaaggtatactgatggagg-3′ and 5′-aaggaaccccttgccccgctcacgtgcacatctctttcctgtggcaaacg-3′. This resulted in drug resistance-conferring cassettes flanked upstream by sequence corresponding to bp −43 to +16 bp of Tb*GAPDHL* (encoded by Tb927.9.9820) and downstream by 59 bp matching sequence from bp 11–69 beyond the stop codon for Tb*GAPDHL*. For PCR from genomic DNA isolated from Tb*GAPDH* ^+/−^ cell lines a primer combination of 5′-gcatggacaataatggtcgg-3′ and 5′-cgacattctttcccagtacc-3′ was used, resulting in amplification of drug resistance-conferring cassettes flanked by 558 bp of upstream and 389 bp downstream homology targeting flanks; these PCR products were used for transfection of Tb*GAPDH* ^+/−^ cell lines.

For Southern transfers, restriction endonuclease-digested genomic DNA was blotted to Hybond-N (GE Healthcare); blots were hybridized against DNA sequences corresponding to either coding sequence for Tb*PGKL* or Tb*GAPDHL* or 3′ intergenic sequence. DNA probes were produced using an AlkPhos Direct Labelling Kit. This, and detection with CDP-Star (GE Healthcare), were carried out according to the manufacturers' instructions. Coding sequence for Tb*GAPDHL* was amplified using a primer combination 5′-ccttgcctatacccataggt-3′ and 5′-atggctgtgtacgagcaatc-3′; 3′ intergenic sequence amplified using a primer combination 5′-cgctacatgactaccagaatgc-3′ and 5′-ccatatgttcgtgtggtacg-3′. For Tb*PGKL* coding and intergenic sequences were amplified using primer combinations 5′-atgtctcttagcgccttacg-3′ and 5′-aacgattggtgagttcacgc-3′ or 5′-gcaggcgttgatgagatcat-3′ and 5′-ctattcaccaactgttgcgc-3′, respectively.

## Results and Discussion

### Degeneracy of trypanosome GAPDH- and PGK-like proteins

*T. brucei* genes Tb927.9.9820 (Gene identification numbers as given in TriTrypDB Version 6.0 [25]) and Tb927.11.2380 encode proteins homologous to the glycolytic enzymes GAPDH and PGK, respectively. Proteomic analyses indicate constitutive expression of both Tb927.9.9820 and Tb927.11.2380 in the lifecycle stages amenable to cell culture (procyclic and bloodstream stages). Genes orthologous to Tb*GAPDHL* (standing for GAPDH-like) are conserved in all trypanosomatid species for which genome sequences are available. Tb*PGKL* (PGK-like) orthologs are present in *Trypanosoma* species for which genome sequences are available, but in *Leishmania* species only *PGKL* pseudogenes are evident (*e.g.* LmjF27.1720 in *L. major* strain Friedlin).

**Figure 1:**
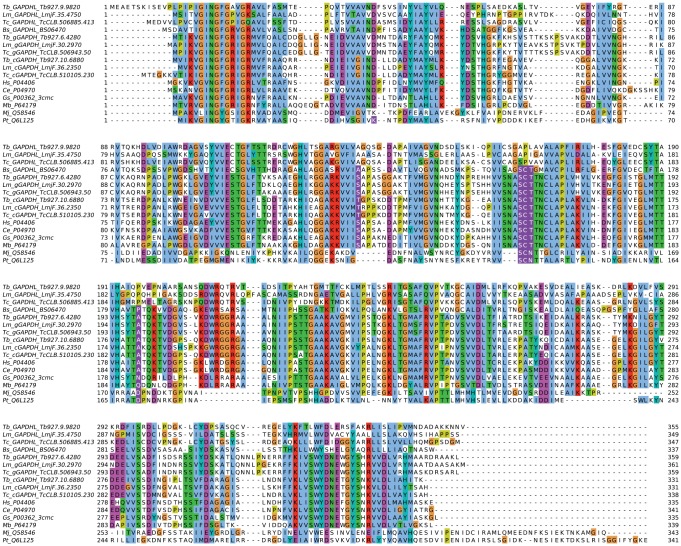
Sequence alignment of trypanosomatid GAPDHL proteins with authentic GAPDH. The alignment was built using MUSCLE and sequences are named with species abbreviations (Tb =  *T. brucei*, Tc =  *T. cruzi*, Lm =  *L. major*, Bs =  *B. saltans*, Gt =  *Geobacillus stearothermophilus*, Mb =  *Mycobacterium bovis*, Hs =  *Homo sapiens*, Ce =  *Caenorhabditis elegans*, Mj =  *Methanocaldococcus jannaschii*, Pt =  *Picrophilus torridus*) followed by a locus code (kinetoplastid sequences) or UniProt accession. For kinetoplastid sequences, cGAPDH indicates the cytosolic isoform and gGAPDH the glycosomal enzyme. Only one each of the tandem copies of gGAPDH is shown for each trypanosomatid. Residues mentioned in the text are highlighted as white on purple.

**Figure 2:**
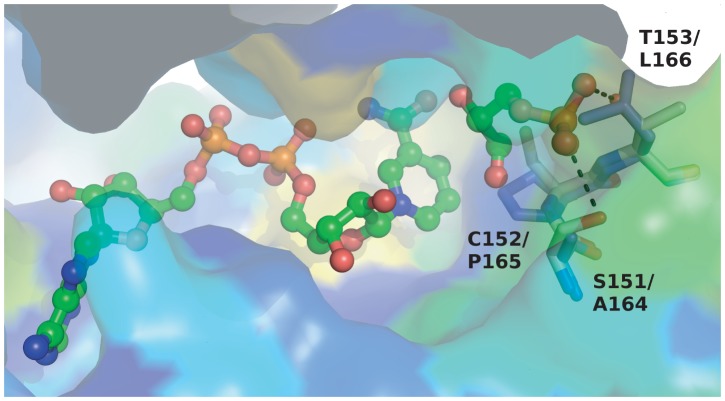
Surface representation of TbGAPDHL with substrates. The representation is colored according to sequence conservation (blue-red, low to high) by Consurf. The catalytic Cys residue, neighboring residues (white sticks) and bound ligands (covalently attached glyceraldehyde-3-phosphate and NAD; ball and stick) from superimposed *G. stearothermophilus* GAPDH (PDB code 3cmc) are shown, with H-bonds illustrated as dotted lines. Corresponding residues in the TbGAPDHL model (purple sticks) are functionally incapable. Residues are labelled as template/model.

We describe trypanosomatid gene products characterized in this work as GAPDH-like and PGK-like because numerous key residues required for substrate binding or catalysis in GAPDH and PGK enzymes across the breadth of evolution are not conserved. These substitutions were placed in a structural context using protein models and predictions made of which, if any, of the canonical functions remained. For *Tb*GAPDHL (Fig. 1), the most significant loss is that of the catalytic, nucleophilic Cys152 residue which is replaced by Pro. Fig. 2 shows a comparison of the TbGAPDHL model with the structure of *Geobacillus stearothermophilus* GADPH bound to substrates (PDB code 3cmc; [54]). Mutation of this Cys residue to Ala in the bacterial enzyme leads to loss of activity [55]. The predicted loss of activity is also strengthened by the loss, in *Tb*GAPDHL and most trypanosomatid orthologs, of the potential for hydrogen bonds contributed by conserved flanking residues Ser and Thr (numbered 151 and 153, respectively in the template) which are replaced by Ala and Leu, respectively, in *Tb*GAPDHL (Fig. 2). Curiously, the *Tb*GAPDHL model indicates that the cofactor-binding pocket retains a similar size and shape to that in active enzymes, raising the possibility that NAD^+^ or a similar compound might still be bound in *Tb*GAPDHL. Although the power of sequence conservation mapping for revealing functional sites is limited by the small number of trypanosomatid GAPDHL sequences available, the region corresponding to the cofactor adenosine site in the bacterial enzyme is surrounded by a number of conserved residues in the GAPDH-like group, including the GINGFG region from 18–23 (*T. brucei* numbering; Fig. 1). This raises a tantalizing possibility that the cofactor site in a clearly inactive *Tb*GAPDHL has been retained for binding a ligand. Finally, amino acid conservation between GAPDHL orthologs present in different trypanosomatids is very much lower than the conservation seen between the catalytically active GAPDH isoforms found in the glycosomes and cytosol of different trypanosomatids (Table 1). This also strongly suggests that GAPDHL proteins do not retain a catalytic activity.

**Table 1:** Range and mean percentage amino acid identities between GAPDH(-like) groups in trypanosomatids.

	GAPDHL	Cytosolic ‘GAPDH’	Glycosomal GAPDH
**GAPDHL**	29–95 (52)	-	-
**Cytosolic ‘GAPDH’**	21–32 (25)	76–96 (85)	-
**Glycosomal GAPDH**	23–28 (25)	52–57 (55)	79–96 (87)

Each group contains sequences (eliminating tandem duplicates) from *T. brucei, T. cruzi, T. vivax, L. braziliensis, L. mexicana, L. major, L. infantum, L. tarentolae*, and *Endotrypanum monterogeii*.

**Figure 3:**
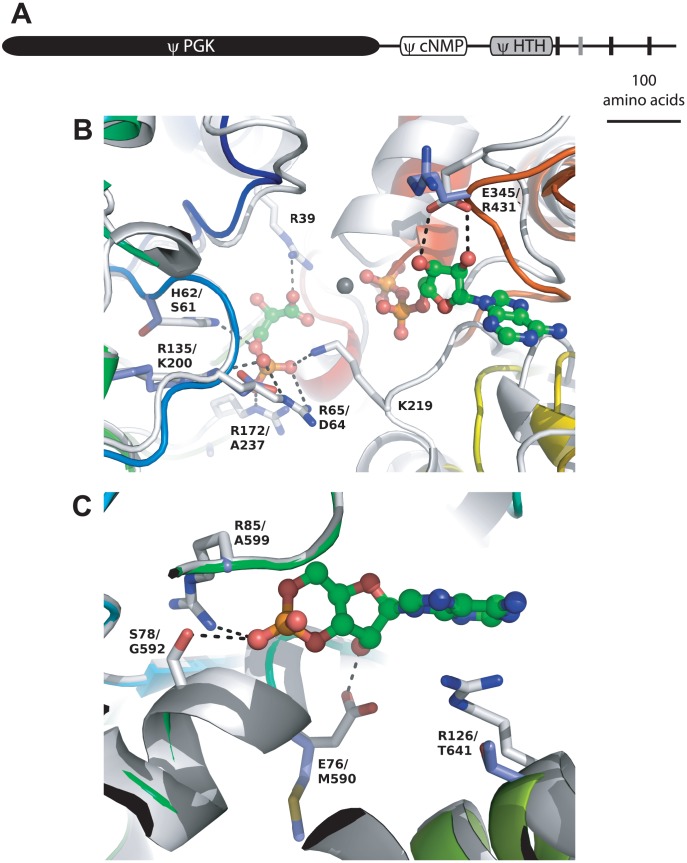
Modular architecture and modelling of *T. brucei* PGKL proteins. (**A**) Cartoon schematic illustrating the modular architecture of TbPGKL. Positions of the predicted membrane-spanning helices are shown as vertical black bars; the additional helix predicted by Phobius is denoted by the grey vertical bar. (**B**) Comparison of the *Tb*PGKL ‘PGK’ domain model and the *T. brucei* PGK template structure (PDB code 13 pk; [56]) in the vicinity of the catalytic site. The template is shown as white cartoon with key binding residues shown as sticks. Bound ligands (ADP, 3-phosphoglycerate) are shown as ball-and-stick, bound Mg^2+^ as a grey sphere: their hydrogen bonds with the protein are shown as dotted lines. The model cartoon is colored from blue to red (N- to C-terminus) and equivalent residues to those shown for the template as purple sticks. Residues are labelled as template/model. In a few cases there is no equivalent model residue due to deletions in the alignment. (**C**) Comparison of the *Tb*PGKL post-‘PGK’ domains model and the *T. thermophilus* CRP template structure (PDB code 4ev0; unpublished) in the vicinity of the cAMP binding site. The template is shown as white cartoon with key binding residues shown as sticks. Bound cAMP is shown as ball-and-stick: its hydrogen bonds with the protein are shown as dotted lines. The model cartoon is colored from blue to red (N- to C-terminus) and equivalent residues to those shown for the template as purple sticks. Residues are labelled as template/model.

*Tb*PGKL exhibits an unusual modular architecture (Fig. 3A): the N-terminal ‘PGK’ domain is followed by a domain homologous to cyclic nucleotide (cNMP) binding proteins (Pfam entry *cNMP_binding*; PF00027 (TbPGKL numbering), then a region matching helix-turn-helix (HTH) DNA binding proteins (*e.g. HTH_Crp_2*; PF13545). The remaining C-terminal 165 residues contain no recognizable domain but predictors suggest the presence of three (TMHMM prediction) or four (Phobius) transmembrane helices. A lack of catalytic activity is as equally clear for PGKL as it is for GAPDHL (Fig. 3B). A model of the TbPGKL ‘PGK’ domain based on the best available template, *T. brucei* PGK (PDB code 13 pk; [56]), reveals perturbations to important interactions with both substrates (Fig. 3B). At the phosphoglycerate binding site, Arg39 which makes a key interaction with the carboxylate group [56] is aligned with a deletion in TbPGKL and no suitable replacement residue is seen in the model. Similarly, the *T. brucei* PGK structure shows that the 3-phospho group is electrostatically bound by five positively charged side chains His62, Arg65, Arg135, Arg172 and Lys219. Only one corresponding position is occupied by a basic residue in *Tb*PGKL (Lys200 aligned with Arg135) and an acidic residue, Asp64 replaces Arg65 of the catalytically active trypanosome PGK. The ATP binding site in active PGKs is generally not conserved in *Tb*PGKL, for example losing Glu345 which makes twin H-bonds to the ribose ring of bound ATP, this being replaced by Arg in *Tb*PGKL in a region that additionally is subject to a one-residue deletion. Modeling suggests there are no definitive steric impediments to binding of a ligand to *Tb*PGKL in the equivalent pocket to the ATP site but, even among the PGKL sequences from four *Trypanosoma* species, residues lining the pocket are not conserved. As with GAPDHL, poor inter-species conservation across the PGK domain in PGKL orthologs, relative to catalytically active PGK isoforms in trypanosomatids, or indeed other organisms, suggests PGKL proteins do not exhibit catalytic activity.

A model was also built of the two domains C-terminal to the catalytic domain. In order to assess implications for cyclic nucleotide binding, the top-scoring cAMP-bound structure, of *Thermus thermophilus* CRP (PDB code 4ev0; unpublished) was used as template. Again, key residues that hydrogen bond the cyclic nucleotide (Glu76, Ser78, Arg85) or form other substantial interactions with it (Arg 126) are missing in the PGKL model (Fig. 3C). A structure-based prediction of DNA-binding ability [57] strongly suggests that the region homologous to DNA-binding proteins in PGKL no longer has that capacity. While the DNA-binding region of *T. thermophilus* CRP scored 1.90, above even a stringent (5% predicted false positive) threshold of 1.30, the corresponding domain of PGKL scored only −0.34. Curiously, with the exception of the PGKL proteins described here, the combination of cNMP-binding and helix-turn-helix domains is otherwise only seen in bacterial proteins, and is characteristic of the family of bacterial transcription factors exemplified by cAMP receptor proteins [58]. There is also additional novelty, albeit enigmatic, to the linkage of degenerate PGK and cyclic nucleotide-binding domains because whilst Pfam shows hundreds of different domain architectures including a cyclic nucleotide binding domain, these do not currently include fusions with glycolytic enzymes (although a single bacterial sequence (UniProt ID Q0F2T2) indicates C-terminal fusion of the pentose phosphate pathway enzyme glucose-6-phosphate dehydrogenase to a cyclic nucleotide binding domain).

### Flagellar localizations of *Tb*GAPDHL and *Tb*PGKL

**Figure 4:**
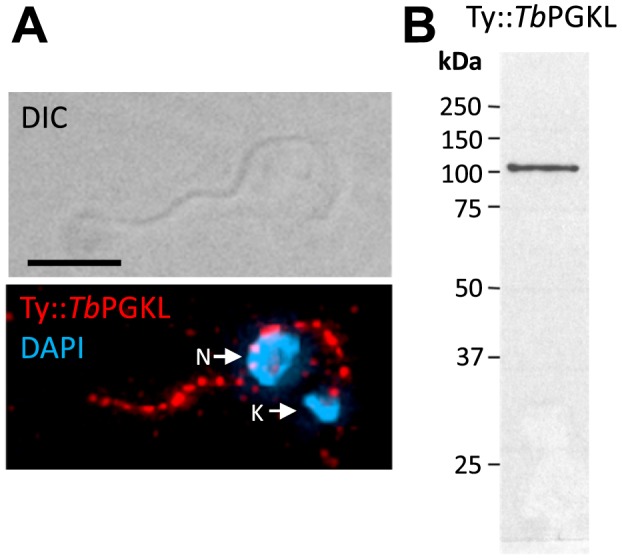
Flagellar localization of *Tb*PGKL. (**A**) Indirect immunofluorescence using monoclonal antibody BB2 reveals axonemal localization of Ty::*Tb*PGKL in detergent-extracted procyclic *T. brucei* cytoskeletons. Cytoskeletons were stained with 4′,6-diamidino-2-phenylindole (DAPI) to detect mitochondrial (kinetoplast, K) and nuclear (N) DNA. The inset shows how the indirect immunofluorescence signal extends close to the kinetoplast, consistent with axoneme association. Scale bar denotes 5 µm. (**B**) Immunoblot analysis of detergent- and NaCl-extracted flagella isolated from procyclic cells expressing Ty::*Tb*PGK-like protein using BB2 detects a single band of the expected molecular mass.

**Figure 5:**
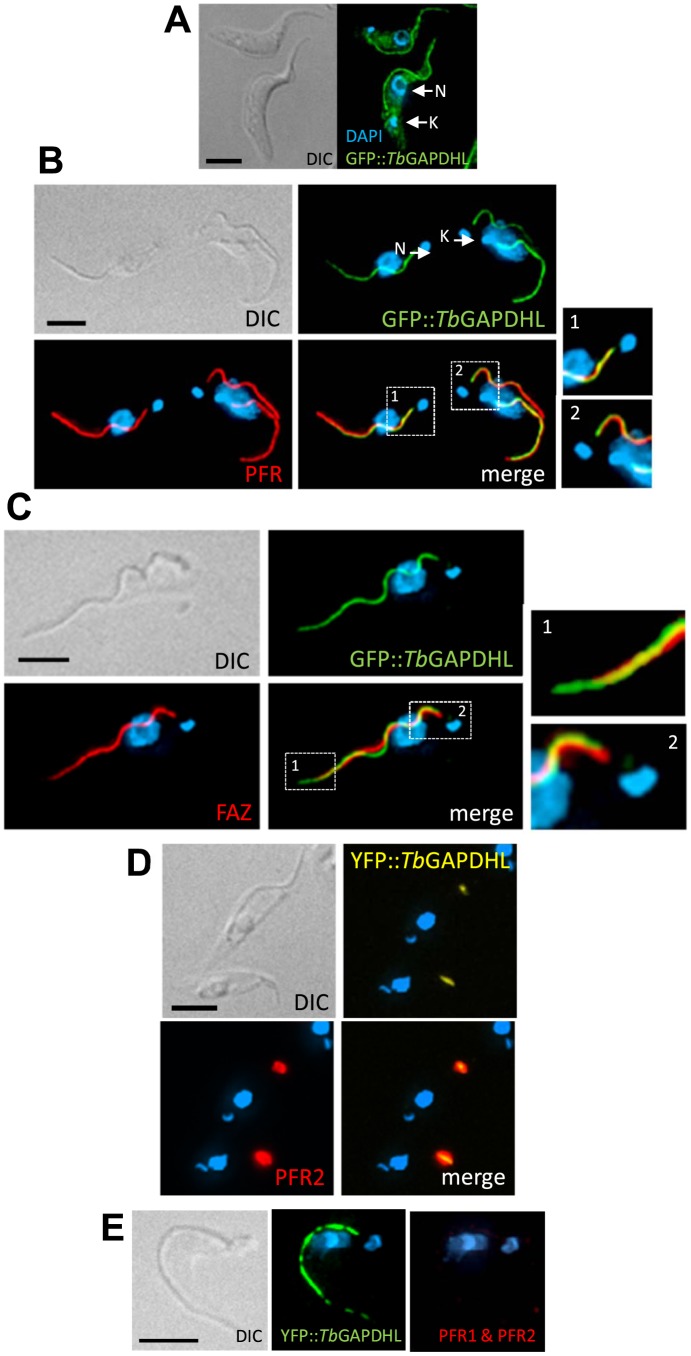
PFR localization of *Tb*GAPDHL. (**A**) Localization of GFP::*Tb*GAPDHL in procyclic *T. brucei* cells. (**B**) Indirect immunofluorescence of detergent-extracted cytoskeletons using the monoclonal antibody L8C4 to detect the major PFR protein PFR2 suggests GFP::*Tb*GAPDHL is a novel PFR component. Insets 1 and 2 indicate that at the proximal end of the flagellum PFR2 incorporation into flagellar skeleton does not begin prior to GFP::*Tb*GAPDHL incorporation – *cf* inset 2 in (**C**). (**C**) A lack of co-localization in indirect immunofluorescence of cytoskeletons using the monoclonal antibody L3B2 indicates GFP::*Tb*GAPDHL is not a cytoplasmic FAZ component: inset 1 indicates flagellar GFP::*Tb*GAPDHL fluorescence extends beyond the end of the cell body as denoted by L3B2 labelling of the cytoplasmic FAZ filament; inset 2 highlights how assembly the cytoplasmic FAZ filament detected by L3B2 initiates before assembly of GFP::*Tb*GAPDHL into the flagellar architecture. (**D**) Change in YFP::*Tb*GAPDHL localization in Tb*CaM* RNAi mutants: following RNAi induction and failure of PFR assembly YFP::*Tb*GAPDHL co-localizes with aggregates containing PFR2 protein (detected by indirect immunofluorescence with monoclonal antibody L8C4). (**E**) PFR localization of GFP::*Tb*GAPDHL is retained in *snl-2* RNAi mutants; detergent extracted cytoskeletons were also stained for indirect immunofluorescence with L13D6 to highlight failure to incorporate either PFR1 or PFR2, the two major PFR components, into the flagellar architecture. DIC, differential interference contrast; N, nucleus; K, kinetoplast. Scale bars denote 5 µm.

Neither *Tb*GAPDHL nor *Tb*PGKL contain predicted signal peptides or N-terminal mitochondrial leader sequences. Thus, both were expressed as N-terminal fusions to either GFP or YFP (*Tb*GAPDHL) or a single, 10 amino acid Ty-epitope (*Tb*PGKL) from endogenous chromosomal loci; the endogenous locus tagging approach maximizes the possibility that gene expression levels of the tagged proteins is comparable to that of the native proteins. Both GFP::*Tb*GAPDHL and Ty::*Tb*PGKL localized to the flagellum, and their retention in detergent-extracted cytoskeletons indicated tight association with the structural architecture of the flagellum (Figs. 4–5). In trypanosomes, a complex series of filaments spanning outer and inner mitochondrial membranes attach the mitochondrial genome (or kinetoplast) to the flagellar basal body from which the axoneme extends [59]. The punctate indirect immunofluorescence signal from cytoskeletons decorated with the monoclonal antibody BB2 (to detect the Ty-epitope of Ty::*Tb*PGKL) extended close to the kinetoplast (Fig. 4A), indicative of axonemal association. Although predicted to be an integral membrane protein, the association of *Tb*PGKL with detergent- and salt-extracted axonemes should not be viewed as surprising since other integral flagellar membrane proteins in other organisms are also retained in detergent-extracted flagella (*e.g.* PKD2 in *C. reinhardtii* [60]). Detection of Ty::*Tb*PGKL in detergent- and NaCl-extracted flagella also points to a high-affinity interaction between Ty::*Tb*PGKL and an, as yet, unknown axonemal component (Fig. 4B). An absence of detectable Ty::*Tb*PGKL on the microtubules of the sub-pellicular microtubule corset in cytoskeletal preparations provides further indication of the specificity of the Ty::*Tb*PGKL-axoneme interaction.

GFP::*Tb*GAPDHL localized to the flagellum and to a lesser extent the cytosol, too (Fig. 5A). In detergent-extracted cytoskeletons, GFP::*Tb*GAPDHL localized only to the flagellum, but in neither whole cells nor cytoskeletons did the GFP fluorescence extend as close to the kinetoplast as the indirect immunofluorescence signal from Ty::*Tb*PGKL, suggesting localization of *Tb*GAPDHL to either the paraflagellar rod (PFR) or flagellum attachment zone (FAZ). The PFR is an elaborate, filamentous extra-axonemal structure restricted in its evolutionary distribution to trypanosomatids and other protists from the phylum Euglenozoa (*e.g. Euglena gracilis*) [50], and it is built from two abundant proteins (PFR1 and PFR2) plus a number (∼30) of less abundant components. In *T. brucei*, the PFR is assembled from the point where the flagellum exits its flagellar pocket to emerge onto the cell surface – *i.e.* ∼2 µm distal to the basal body. In addition to its essentiality for cell motility [61], the *T. brucei* PFR is also important for ensuring the flagellum remains securely attached to the cell body via filaments that connect the structural architecture of the flagellum to the FAZ [47]. Thus, to distinguish between possible PFR and FAZ localizations, we first compared the fluorescence pattern from YFP::*Tb*GAPDHL with indirect immunofluorescence signals from the monoclonal antibodies L8C4, which recognizes PFR2, and L3B2, which recognizes FAZ1 protein from the cytoplasmic face of the FAZ. Fluorescence signals from detergent-extracted cytoskeletons clearly revealed YFP::*Tb*GAPDHL co-localized with PFR2 and was absent from the cytoplasmic face of the FAZ, as defined by L3B2 labelling (Fig. 5B–5C).

At first glance, co-localization of *Tb*GAPDHL with PFR2 is perhaps surprising since it is not a component of the published PFR proteome [62]. However, that proteome was derived from comparisons between flagella isolated from wild-type procyclic cells and Tb*PFR2* RNAi mutants that, due to *Tb*PFR2 loss, build only a rudimentary PFR, which is sufficient to connect the axoneme through to the cytoplasmic face of the FAZ filament, but cannot serve its normal function in motility [48], [61]. This rudimentary structure lacks the characteristic elaborate three-domain lattice-like organization of the normal (PFR2-containing) PFR, and is deficient in approximately 30 known or candidate PFR proteins. Recently, we reported that RNAi against a PFR-localized isoform of calmodulin (*Tb*CAM, encoded by a cluster of four identical, tandem duplicated genes (Tb11.01.4621–Tb11.01.4624) resulted in a complete failure of PFR assembly. Normally, this calmodulin isoform is found in (a) connections linking the PFR to outer-doublet microtubules four-to-seven of the axoneme, (b) the proximal, intermediate and distal zones of the PFR, and (c) fibrous connections linking the PFR to the cytoplasmic FAZ filament, but following *Tb*CAM RNAi induction even the connecting links between PFR and axoneme are seldom built. In the absence of even a rudimentary PFR, no connection from the axoneme through to the cytoplasmic FAZ filament is seen [47]. To determine whether *Tb*GAPDHL is present within the innermost proximal region of the PFR (which is still assembled at least to some degree in Tb*PFR2* RNAi mutants), connections between PFR and axoneme, or the connection between PFR and the cytoplasmic FAZ filament, we compared the localizations of YFP::*Tb*GAPDHL in Tb*CAM* and Tb*PFR2* RNAi mutants. We reasoned that if *Tb*GAPDHL is incorporated into the proximal zone of the PFR or helps mediate any of the afore-mentioned connections, then normal YFP::*Tb*GAPDHL localization would be observed in Tb*PFR2* RNAi mutants, but lost following induction of Tb*CAM* RNAi. As shown in Fig. 5D–5E, this was the case. Co-localization of YFP::*Tb*GAPDHL with a ‘blob’ of *Tb*PFR1 and *Tb*PFR2 protein in the induced Tb*CAM* RNAi mutant reflects the transport into the flagellum of PFR proteins, and then the aggregation of these proteins, albeit into a structure lacking the ornate form seen normally [47]. We discussed previously [47] how retention of PFR components in detergent-extracted Tb*CAM* RNAi mutants is likely due to the deployment of a much reduced amount of calmodulin protein produced following RNAi for the assembly of axoneme-PFR links: typically the assembly of these connections occurred at the flagellar pocket exit point (where the PFR is first assembled), at the anterior cell end which is the last point of connection between the flagellum and the cell body (as shown in the images in Fig. 5D) or at the distal end of the flagellum.

### Generation of Tb*GAPDHL* and Tb*PGKL* null mutants

**Figure 6:**
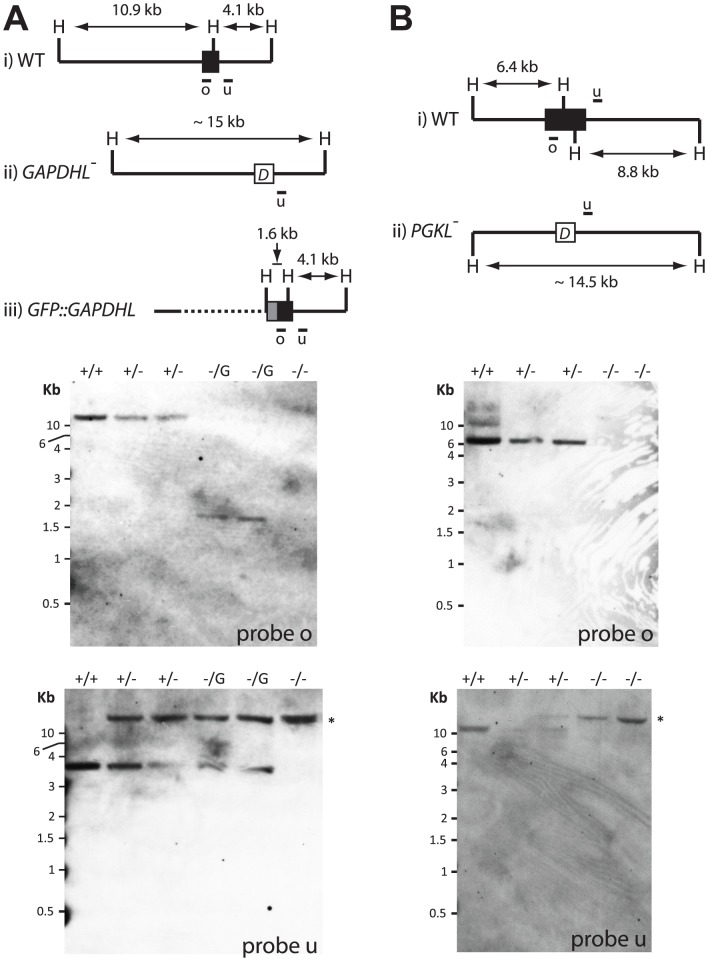
Generation of Tb*GAPDHL* and Tb*PGKL* procyclic null mutants. (**A**) Generation of Tb*GAPDHL* null mutants. (**B**) Generation of Tb*PGKL* null mutants. Cartoon schematics denote gene loci annotated with HindIII (H) restriction sites for (i) wild-type loci; (ii) loci following gene disruption and (iii) following endogenous gene-tagging with GFP (Tb*GAPDHL* only). Southern analysis of genomic DNA digested overnight at 37°C with HindIII shows blots probed sequentially with either coding sequence from the targeted gene (probe o) or sequence from the 3′ intergenic region (probe u). Relative positions of the probes are shown in the cartoon schematics. In (**A**) the order of lanes is 1, wild-type *GAPDHL* ^+/+^ *T. brucei*; 2, heterozygous *GAPDHL* ^+/−^ cells resistant to phleomycin; 3, heterozygous *GAPDHL* ^+/−^ cells resistant to blasticidin/HCl; 4-5, *GAPDHL* ^+/−^ heterozygotes from lanes 2 and 3, respectively, in which endogenous tagging of the remaining wild-type allele results in expression of a recombinant e*GFP:GAPDHL*; 6, a *GAPDHL^−/−^* mutant obtained from the stable transformation of the phleomycin-resistant heterozygote cells from lane 2. In (**B**) the order of lanes is 1, wild-type *PGKL* ^+/+^, 2, heterozygous *GAPDHL* ^+/−^ cells resistant to phleomycin; 3, heterozygous *GAPDHL* ^+/−^ cells resistant to blasticidin/HCl; 4–5, independently obtained *PGKL* ^−/−^ mutants derived from the stable transformation of heterozygous cell lines analyzed in lanes 2 and 3, respectively.

We looked at the essentiality of Tb*GAPDHL* and Tb*PGKL* in procyclic *T. brucei* by sequentially replacing both alleles of each gene (*T. brucei* is diploid) with genes conferring resistance to either phleomycin or blasticidin S in order to create ΔTb*GAPDHL* and ΔTb*PGKL* mutants. Following isolation of gDNA from stably transformed cell lines, Southern blot analyses were used to confirm the generation of Tb*GAPDHL* and Tb*PGKL* null mutants (Fig. 6). Both ΔTb*GAPDHL* and ΔTb*PGKL* cells grew normally without detectable morphological or motility defects. Thus, neither Tb*GAPDHL* nor Tb*PGKL* are essential in procyclic *T. brucei*, at least under the standard culture conditions used by many groups for growth and genetic manipulation of African trypanosomes. A similar absence of discernable motility or growth phenotypes has been reported by independent labs for procyclic trypanosome RNAi mutants depleted for other axonemal or PFR proteins, including several axonemal proteins that are widely conserved in flagellate eukaryotes [62]–[64]. The current absence of discernable phenotypes for Tb*GAPDHL* and Tb*PGKL* null mutants presumably reflects redundancy associated the structural complexity of the flagellar architecture, function in another life cycle stage and/or a requirement for these proteins under specific environmental conditions yet to be mimicked by the artificial nature of *in vitro* culture using complex media.

### Origin(s) of GAPDHL and PGKL degeneracy?

Conservation of Tb*GAPDHL* (BS06470; gene identification numbers as given in the *B. saltans* gene database available at http://www.genedb.org/Homepage/Bsaltans) and Tb*PGKL* (BS70390) orthologs in *Bodo saltans*, a free-living relative of the trypanosomatids indicates that the origin(s) of *GAPDHL* and *PGKL* pre-dates divergence of the parasitic trypanosomatid family. Intriguingly, the *B. saltans* GAPDHL protein, unlike its trypanosomatid counterparts, retains the hallmarks of catalytic activity including the SCT motif harboring the catalytic Cys residue (Fig. 1). A model of *B. saltans* GAPDHL (not shown) revealed only minor changes to the set of key substrate-binding residues and no insertions or deletions in the region that would change the shape of the active site. A few interactions in the *Geobacillus stearothermophilus* GAPDH are absent in the *B. saltans* model: *e.g.* the hydrogen bond between Ser120 in the former and NAD^+^ cannot be made by the Ala residue of the latter; likewise the hydrogen bond between *G. stearothermophilus* Asn183 and NAD^+^ cannot be made by the Pro substituted in *B. saltans* GAPDHL. However, these changes are minor and Fig. 1 shows neither Ser120 nor Asn183 is universally conserved among prokaryote GAPDH enzymes. Importantly, this interpretation dates the loss of activity in GAPDHL proteins to after divergence of the parasitic trypanosomatids from their free-living ancestors. In contrast, the predicted *B. saltans* PGKL protein not only exhibits the same overall architecture as the *Trypanosoma* proteins, but shares the non-conservation of key active-site residues.

Classically, adaptations to obligate parasitism are associated with the streamlining of gene content. Indeed, comparative analyses of metabolic repertoires within the trypanosomatid family reveal extensive metabolic streamlining has occurred repeatedly following the radiation of the different trypanosomatid lineages from a common ancestor, presumably as a consequence of niche adaptation [65]–[67]. Thus, although our procyclic mutants null for either Tb*GAPDHL* or Tb*PGKL* present no discernable phenotype, we predict retention and expression of these genes confers a fitness benefit during at least one stage of the complex, natural trypanosome transmission cycle, and potentially speaks to the environmental challenges the digestive tract of the tsetse fly, as opposed to liquid culture, is likely to pose for the motility and migration of *T. brucei* during its developmental cycle in the vector [68]. Clearly, in the case of *Leishmania* species, however, any necessity for a PGKL protein was lost. One notable difference between the biology of the *Trypanosoma* species and *B. saltans* versus *Leishmania* is that in the former flagella emerge onto the cell surface and remain stably attached to the plasma membrane – in the case of the biflagellate *B. saltans* the recurrent flagellum attaches to the plasma membrane [69] – whereas in *Leishmania* the flagellum is free from the cell body following emergence from its flagellar pocket and only a very small FAZ-like region of adhesion is evident as the flagellum exits its pocket [70]. However, the generation of procyclic Δ*TbPGKL* cells indicates that if *Tb*PGKL is an integral component of the FAZ, then it is non-essential, at least in cultured trypanosomes.

There are numerous examples of proteins with degenerate ‘enzymatic’ domains that function in diverse cellular contexts, including examples from trypanosomatids (*e.g.* [71]–[73]). Yet, the presence in the *T. brucei* flagellum of degenerate-looking versions of enzymes that function sequentially within the glycolytic pathway appears unlikely to be a coincidence. We suggest Tb*GAPDHL* and Tb*PGKL* provide molecular evidence for degeneration of a flagellum-localized partial glycolytic pathway during kinetoplastid evolution.

Flagellar motility is critically dependent upon the coordinated activity of multiple classes of axonemal dynein ATPases. Discoveries in taxonomically diverse protists of flagellum-localized isoforms of diverse enzymes classically associated with ATP production and homeostasis is highly suggestive of significant compartmentalized energy provision within the eukaryotic flagellum, at least under specific environmental conditions [74]. Degeneration of a partial flagellar glycolytic pathway during kinetoplastid evolution could simply be explained as a consequence of niche adaptation to an environment with limited glucose availability, but notwithstanding the reduced availability of carbohydrates in some of the lifecycle niches occupied by flagellate *Leishmania* and *T. brucei*, this seems unlikely given (a) the near ubiquity of glycolysis as a major catabolic pathway in eukaryotes and (b) the cosmopolitan distribution of kinetoplastids in nature. Use of the PFR as a scaffold into which adenylate kinase isoforms are anchored [75], [76] could have provided an adaptation that resulted in loss of a flagellar glycolytic pathway, although in other flagellates flagellum-localised isoforms of adenylate kinases and glycolytic enzymes coexist [74]. Alternatively, the absence from extant trypanosomatids of conserved regulatory mechanisms which control glycolytic flux in other organisms appears to be a consequence of the exclusive re-compartmentalization of glycolytic enzymes from the cytosol to peroxisomes that took place during kinetoplastid evolution [10], [14], [16], [17], [19]. Changes to glycolysis regulation and compartmentalization during kinetoplastid evolution could therefore have provided the necessary selective pressure for loss from the flagellum, or indeed other cellular compartments, of enzymes involved in catabolism of glucose to its glycolytic intermediate 3-phosphoglycerate. In that regard, it is noteworthy that a *T. brucei* hexokinase isoform (HXK2) dually located in glycosomes and the flagellum [77] is itself catalytically inactive unless co-expressed with the paralogous HXK1, wherein a hexameric recombinant enzyme with kinetic properties similar to native hexokinase purified from *T. brucei* cells is reconstituted [78]. Tb*HXK2* is paralogous to Tb*HXK1*, suggesting recent gain of a cytoskeletal function for an abundant trypanosome glycolytic enzyme. In contrast, phylogenetic analysis (see Methods for further details) provides no evidence that either GAPDHL or PGKL evolved following paralogous duplication of genes encoding glycosomal GAPDH or PGK.

In the case of PGKL, its unusual modular architecture suggests degeneration of a flagellar PGK isoform. In contrast, the origin of a catalytically degenerate GAPDHL is more complex to explain, and conceivably speaks directly to an emerging view that many ubiquitous proteins inside cells are multifunctional [79]. Thus, glycolytic enzymes, notably GAPDH [80], [81], provide prime examples for the paradigm of protein moonlighting – a now commonly recognized phenomenon whereby many proteins function in cellular processes unrelated to the role(s) for which they were originally characterized and are better known [79]. Various metabolic enzymes, including GAPDH, form intracellular filaments in response to a range of cues [81]–[83], and the PFR is a complex filament-based structure that assembles following IFT-dependent transport of its component parts to the flagellar distal tip [84], [85]. Filament-forming properties of abundant soluble enzymes, such as GAPDH, may therefore have been a critically exploitable feature during the evolution of PFR structure. A structural role for GAPDH in PFR assembly is also compatible with dual functionality in helping support, at some point in kinetoplastid evolution, a partial flagellar glycolytic pathway. Indeed, previous reports illustrate the importance of the PFR as a platform for metabolic activities [76], [86].

*B. saltans* is the closest free-living relative of the trypanosomatid family for which a publicly accessible genome sequence is available. The likelihood that the *B. saltans* ortholog of trypanosomatid GAPDHL proteins is catalytic active suggests degeneracy of the latter occurred relatively recently. Cytosolic and glycosomal GAPDH activities have been described in the kinetoplastid *Trypanoplasma borelli* [87], and different trypanosomatids, including *T. brucei* and some *Leishmania* species [88], [89]. Yet, no ortholog of the trypanosomatid enzyme responsible for cytosolic GAPDH activity is evident within the *B. saltans* genome (our unpublished observation), consistent with a suggestion that cytosolic trypanosomatid GAPDH owes its origin to a lateral gene transfer after the divergence of a trypanosomatid ancestor from other kinetoplastid lineages [87]. If dual localization of TbGAPDHL to cytosol and flagellum (Fig. 5A) is not an artefact of gene-tagging, then relatively recent arrival of a laterally transferred cytosolic GAPDH could have supplanted the ancestral enzymatic function(s) of GAPDHL, resulting in degeneration of an active catalytic site and leaving a dual located protein in possession of only its (still enigmatic) flagellar function. Curiously, in *L. donovani*, gene knockout of cytosolic GAPDH results in reduced infectivity of visceral organs in a mouse model [89], yet in some *Leishmania* species cytosolic GAPDH is either present as a pseudogene (*e.g.* in the Old World species *L. major*) or absent entirely (*e.g.* in New World *L. braziliensis*), indicating a necessity for cytosolic GAPDH has been lost [89]. From the perspective of our initial characterization of GAPDHL, intriguing data pertaining to cytosolic GAPDH function in *Leishmania* provide further support for our assertion that the retention of GAPDHL orthologs in diverse trypanosomatids is indicative of an important function at some point within these parasites' complex developmental cycles.

Our characterization of *Tb*GAPDHL and *Tb*PGKL adds to an emerging theme that across the breadth of eukaryotic evolution the sub-cellular compartmentalization of glycolytic enzymes is unexpectedly dynamic and complex. Looking across the trypanosomatid family, as well as between trypanosomatids and their more ancestral kinetoplastid relatives, there are species-specific differences in the isoform repertoires of several glycolytic enzymes. Further novelties are also evident within the *B. saltans* genome sequence (*e.g.* a *Bodo*-specific putative PFK isoform, BS33550, possessing a predicted glycosomal PTS-1 targeting signal; our unpublished observations). Such data indicate the glycosomal compartmentalization of glycolytic enzymes and the re-wiring of glycolysis regulation that took place during kinetoplastid evolution occurred against a complex backdrop of paralogous gene duplications and lateral gene transfers. With the advent of next-generation sequencing-led genome surveys of diverse kinetoplastids and other euglenozoan protists underway *e.g.* [90], the sequence data generated from those projects should inform whether our speculations regarding the origins and degeneration of trypanosome PGKL and GAPDHL proteins are correct.
